# Treatment of intractable pruritus with maralixibat in patients with Alagille syndrome before and after reversal of biliary diversion

**DOI:** 10.1002/jpr3.70092

**Published:** 2025-09-30

**Authors:** Chiamaka Nwachukwu, David LeVine, Ryan Himes, John Seal, Bryanna Domenick, Elizabeth B. Rand, Tamir Diamond

**Affiliations:** ^1^ Tulane University School of Medicine New Orleans Louisiana USA; ^2^ Division of Gastroenterology, Hepatology and Nutrition, Ochsner Children's Hospital New Orleans Louisiana USA; ^3^ Louisiana State University Shreveport Louisiana USA; ^4^ Division of Transplant Surgery, Ochsner Health New Orleans Louisiana USA; ^5^ Division of Gastroenterology, Hepatology and Nutrition at Children's Hospital of Philadelphia Philadelphia Pennsylvania USA; ^6^ Department of Pediatrics, Perelman School of Medicine University of Pennsylvania Philadelphia Pennsylvania USA

**Keywords:** cholestasis, ileal bile acid transporter inhibitors, rare pediatric liver disease, serum bile acids

## Abstract

Alagille syndrome (ALGS) is a rare, cholestatic, multisystemic disorder characterized by bile duct paucity. Cholestatic pruritus is a common, and often severe, symptom of ALGS and is the leading cause of liver transplantation. The treatment of cholestatic pruritus is challenging and involves medical and surgical options, such as surgical biliary diversion (SBD) for refractory cases. However, SBD is associated with medical/lifestyle challenges. Maralixibat, an ileal bile acid transporter inhibitor, is a recently approved treatment for cholestatic pruritus in patients with ALGS and is used as part of standard of care. We present cases of two patients with ALGS who initiated treatment with maralixibat: one before, with continuation after, reversal of SBD, and one after SBD reversal. In both cases, treatment with maralixibat was well‐tolerated and demonstrated marked improvements in cholestatic pruritus. This suggests that maralixibat is a pharmacological alternative for patients who would like to pursue reversal of SBD.

## Abstract (highlights)

**What is Known:**

- Cholestatic pruritus is the most common and severe symptom of Alagille syndrome (ALGS).
- Treatment for cholestatic pruritus involves medical and surgical options.
- However, historical medical treatments are used off‐label and are ineffective at controlling symptoms, and surgical options, such as surgical biliary diversion (SBD), are associated with medical and lifestyle challenges.
- Maralixibat was approved for the treatment of cholestatic pruritus in patients with ALGS ≥3 months of age in the United States in 2021 and has since become the standard of care.

**What is New:**

- Maralixibat treatment was safe and demonstrated improvements in cholestatic pruritus in a patient with ALGS with an existing SBD and in a patient with ALGS who had SBD reversal.
- Treatment with maralixibat may be utilized in patients with ALGS who have undergone SBD.

## 1 INTRODUCTION

Alagille syndrome (ALGS) is a rare, autosomal dominant, multisystemic disorder characterized by chronic cholestasis due to a paucity of intrahepatic bile ducts and systemic accumulation of bile acids, resulting in severe pruritus.^1^, ^2^ Cholestatic pruritus is one of the most common ALGS symptoms and is the leading cause of liver transplantation.^3^, ^4^ Typically, cholestatic pruritus presents in children with ALGS at 6–14 months of age and in 15%–45% of cases is severe.^3^, ^4^ Cholestatic pruritus in patients with ALGS is associated with significant patient and caregiver burden; it is often debilitating and affects the patient's quality of life, sleep, and daily activities.^3^, ^5^, ^6^ Additionally, caregivers of patients with cholestatic pruritus report anxiety/depression as well as disruptions in sleep.^7^

Management of pruritus in patients with ALGS is challenging and requires multimodal medical treatment, although, unapproved medications and topical ointments are often ineffective at controlling symptoms in approximately 40% of patients.^4^, ^8^ Historically, in patients who are nonresponsive to systemic/topical treatments, surgical interventions, including surgical biliary diversion (SBD), were used as salvage therapy before liver transplantation. However, these surgical procedures carry a risk of complications.^3^, ^9^, ^10^, ^11^ Even after a technically successful SBD, pruritus may not be alleviated in some patients necessitating liver transplantation, which can result in additional complications and increased risk of mortality.^4^ While cosmetic and psychosocial concerns (e.g., stigma, lifestyle limitations) are typical reasons for SBD reversal, clinical considerations like effective pruritus control, diversion‐related complications, nutritional issues, and improved medical options (e.g., Ileal bile acid transporter [IBAT] inhibitors) can also be important factors and should be considered on a case‐by‐case basis.

IBAT inhibitors are a novel class of recently approved medications considered standard of care for the treatment of cholestatic pruritus in patients with ALGS.^6^, ^7^ Maralixibat was the first IBAT inhibitor approved for the treatment of cholestatic pruritus in patients with ALGS aged ≥3 months in the United States and ≥2 months in the European Union and has been commercially available since 2021.^12^ Maralixibat has demonstrated early and sustained improvements in pruritus, serum bile acid levels, quality of life, and transplant‐free survival for up to 7 years.^13^, ^14^, ^15^, ^16^, ^17^ To our knowledge, there are no published data evaluating the safety and efficacy of maralixibat in patients with breakthrough pruritus following SBD or in those who had reversal of SBD and continue to experience pruritus.

## 2 METHODS

Here, we report on real‐world cases of two patients with ALGS who received maralixibat to treat cholestatic pruritus: one with a SBD who experienced breakthrough pruritus, initiated maralixibat, and subsequently had a reversal of SBD while continuing with maralixibat, and one who had SBD reversal and subsequently received maralixibat.

### 2.1 Ethics statement

Informed consent was obtained for all cases presented in this report as appropriate according to the respective institutions' guidelines.

## 3 RESULTS

### 3.1 Case 1

**Figure 1:**
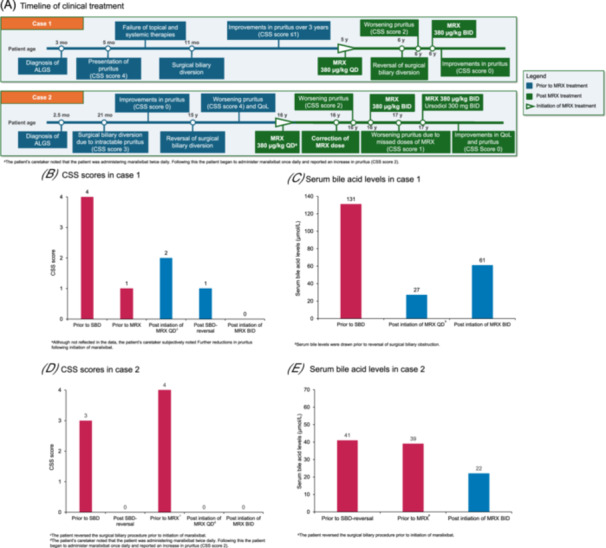
Illustrations of key time points and data pertaining to cases 1 and 2. (A) A timeline of clinical treatment in cases 1 and 2. (B) CSS scores for the patient presented in case 1. (C) Serum bile acid levels for the patient presented in case 1. (D) CSS scores for the patient presented in case 2. (E) serum bile acid levels for the patient presented in case 2. The CSS is a 5‐point scale (0**–**4) where 0 = no scratching, 1 = rubbing or mild scratching when undistracted, 2 = active scratching without abrasions, 3 = abrasions, 4 = cutaneous mutilations, hemorrhage, scarring. ALGS, Alagille syndrome; BID, twice daily; CSS, clinician scratch scale; mo, months; MRX, maralixibat; QD, once daily; QoL, quality of life; SBD, surgical biliary diversion; y, years.

A female patient initially presented at 3 months old with persistent jaundice since birth, difficulty feeding, and failure to thrive (Figure 1A). Initial examination found jaundiced eyes and skin, hepatomegaly, and a 4/6 systolic murmur (Table 1). Laboratory assessments were notable for elevated liver transaminases (alanine transaminase [ALT], 223 U/L; aspartate transaminase [AST], 176 U/L); genetic testing revealed a pathogenic *JAG1* variant. The patient was referred for an in‐hospital specialist evaluation. An echocardiogram demonstrated branch pulmonary artery stenosis; further evaluation with cardiac catheterization revealed severe bilateral pulmonary artery hypoplasia. Chest imaging showed a butterfly deformity of T8, and an abdominal ultrasound found a contracted gallbladder but a normal‐appearing liver. A liver biopsy was performed based on the collective symptoms suggestive of ALGS, which demonstrated a paucity of intrahepatic bile ducts thus confirming the diagnosis. Because the patient was not referred to the current treating physician until much later in their clinical course, it is unclear if the liver biopsy occurred before or after genetic testing.

**Table 1:** Baseline clinical characteristics.

Characteristic	Patient 1	Patient 2
Age,^a^ years	4	16
Sex	Female	Female
Age at ALGS diagnosis	3 months	2.5 months
Age at initiation of pruritus	6 months	12 months
Age at surgical biliary diversion	11 months	21 months
Age at reversal of surgical biliary diversion	5 years	15 years
Genetics	*JAG1* pathogenic variant	*JAG1 heterozygous likely pathogenic variant c.685 T* > *C, p.Cys229Arg*
Facies	Not reported	Broadened forehead, pointed chin, elongated nose with bulbous tip, and triangular face
Heart abnormalities	4/6 systolic murmur, branch pulmonary artery stenosis	Atrial septal defect (closure via Amplatzer device at 4 years old) Heart murmur (mild‐to‐moderate dilation of right atrium and ventricle) Mild left pulmonary artery stenosis
Vertebral abnormalities	Butterfly vertebrae	None
Posterior embryotoxon	Unknown	Not present
Presence of bile duct paucity	Yes	No
Other abnormalities	Contracted gall bladder	Grade 3 renal reflux (at the time of biliary diversion), contracted gallbladder
Antipruritic medications before maralixibat treatment	Naltrexone, rifampicin, ursodiol, hydroxyzine, Eucerin cream	Naltrexone, rifampicin, ursodiol, hydroxyzine

Abbreviation: ALGS, Alagille syndrome.

a At which patient started maralixibat treatment.

At 5 months old, the patient developed severe pruritus (clinician scratch scale [CSS] score 4; Figure 1B) with irritability, blistering, and weeping skin around the groin, associated poor feeding and discomfort due to symptoms. Several oral and topical therapies were trialed, including ursodiol, hydroxyzine, naltrexone, rifampicin, topical nystatin, and petrolatum‐based cream. However, all provided little‐to‐no relief and the pruritus continued to worsen, negatively impacting sleep and quality of life for both the infant and their caregivers. Following failure of the aforementioned therapies, the patient underwent SBD at 11 months of age in an attempt to better control the pruritus.

Following the procedure, the patient experienced sustained improvement of pruritus (CSS score ≤1) for 3 years and required only occasional use of nighttime hydroxyzine. The patient was maintained on ursodiol and occasional nighttime use of hydroxyzine, and all other medications were discontinued. The patient experienced some difficulty achieving durable adherence of an ostomy bag, resulting in skin breakdown. As the patient matured, they became increasingly self‐conscious about the ostomy. Following approval for the treatment of cholestatic pruritus in the United States and positive clinical trial results, the caregiver was consulted on the possibility of augmenting the management of the patient's pruritus with maralixibat. At 5 years of age, the patient was initiated on maralixibat 380 µg/kg once daily, primarily to gauge tolerance, with a plan to revisit options after 1 year of treatment.

After initiating maralixibat, the patient had further improvement in pruritus (CSS score 0), weight gain, and skin appearance. Maralixibat was well tolerated, and the patient did not experience any adverse effects. Due to the demonstrated durable improvement in pruritus after 12 months of maralixibat treatment, the caregiver was given the option to reverse the patient's SBD.

Following reversal of the SBD, the patient reported increased pruritus (CSS score 2) and subsequently at 1‐month post‐reversal, the dose of maralixibat was increased incrementally to 380 µg/kg twice daily. Two months postprocedure follow‐up, the patient's caregiver reported that the patient continued to tolerate the elevated dose and demonstrated sustained improvements in pruritus (CSS score 0) and quality of life (including sleep and appetite). No clinically meaningful differences were observed in change from baseline ALT (baseline: 223 U/L; latest assessment: 179 U/L), AST (baseline: 176 U/L; latest assessment: 129 U/L), or total bilirubin (baseline: 1.0 mg/dL; latest assessment: 1.0 mg/dL) levels. Serum bile acid levels were reduced at the latest assessment compared with baseline (131 vs. 61 µmol/L, respectively; Figure 1C).

### 3.2 Case 2

A female patient presented with a history of jaundice for the first 6 months of life, atrial septal defect, and left pulmonary stenosis (Figure 1A). A liver biopsy at 2.5 months old confirmed a diagnosis of ALGS (Table 1). Genetic testing results identified a likely pathogenic *JAG 1* variant (c.685 T > C, p.Cys229Arg), consistent with the clinical diagnosis of ALGS. At 21 months old, the patient underwent SBD for intractable pruritus (CSS score 3; Figure 1D) to prevent further excoriation of the skin. The patient responded well to the SBD and demonstrated marked improvements in pruritus (CSS score 0). However, due to the social stress related to the presence of an ostomy during adolescence the patient requested reversal of the SBD at age 15 years. The patient subsequently developed severe refractory pruritus (CSS score 4) that began 4 days post‐SBD reversal, resulting in loss of sleep, decline in academic achievements, and self‐mutilation behaviors. The patient was unable to tolerate or did not respond to treatments including rifampicin, ursodiol, hydroxyzine, and naltrexone, and was subsequently initiated on maralixibat 5 months post‐SBD reversal (190 µg/kg once daily for the first 7 days followed by maralixibat 380 µg/kg once daily onwards).

After initiation of maralixibat, the patient reported a marked reduction in pruritus (CSS score 0); however, they experienced nausea due to the taste of the medication, which was alleviated after taking it with apple sauce/Jello. No further nausea was reported and there was no impact on the dose of maralixibat.

Approximately 3 weeks after treatment initiation, the patient's caregiver reported that maralixibat was mistakenly being administered twice daily rather than once daily; following this, the dose of maralixibat was reduced to 380 µg/kg once daily. Subsequently, the patient reported increased pruritus (CSS score 2), and serum bile acid tests showed that levels were still elevated with the once‐daily dose.

At 1.5 months postinitiation of maralixibat, the patient reinitiated maralixibat 380 µg/kg twice daily and subsequently reported only mild pruritus (CSS score 1) and improved sleep. Further follow‐ups highlighted that the patient occasionally missed doses of maralixibat which led to worsening pruritus (CSS score 1), but overall the pruritus was markedly improved while on the medication. Naltrexone, rifampicin, and hydroxyzine were discontinued after 6 weeks on maralixibat therapy.

At 39 months postmaralixibat initiation, the patient reported an overall major improvement in pruritus (CSS score 0), sleep, and quality of life since taking maralixibat consistently. No adverse effects were reported, and the patient was able to also discontinue ursodiol. Slight increases in ALT (baseline: 183 U/L; latest assessment: 23 U/L) and AST (baseline: 138 U/L; latest assessment: 255 U/L) levels were observed but did not impact treatment. Total bilirubin (baseline: 3.8 mg/dL; latest assessment: 2.5 mg/dL) and serum bile acid (baseline: 41 µmol/L; latest assessment: 22 µmol/L; Figure 1E) levels were reduced at the latest assessment compared with baseline.

## 4 DISCUSSION

These case studies demonstrate that administration of maralixibat can be utilized as a “medical diversion” for the treatment of cholestatic pruritus in patients with ALGS in conjunction with SBD or as alternative after SBD reversal. These findings expand the therapeutic scope of maralixibat beyond patients who have not undergone SBD or SBD reversal. In the first case, maralixibat was used as adjunct therapy, enabling successful SBD reversal while maintaining control of pruritus. The second case illustrated maralixibat's utility in managing pruritus after SBD reversal in adolescents with external SBD, particularly when psychosocial stressors, such as ostomy‐related stigma, are present. In both patients, maralixibat led to sustained improvements in pruritus and health‐related quality of life after SBD reversal.

Clinical trials and real‐world studies have established the efficacy of maralixibat in reducing pruritus in patients with ALGS.^13^, ^14^, ^16^ SBD has also shown clinical utility in mitigating cholestasis and its complications including pruritus.^18^ In 2009, Yang et al. reported complete resolution of pruritus in up to 75% of patients with either ALGS or progressive familial intrahepatic cholestasis following partial external biliary diversion for those in whom medical therapy (vitamin supplementation plus ursodiol and/or rifampicin) had previously been ineffective.^18^ However, although SBD may improve pruritus, it has been associated with an increased risk of liver transplant or death.^7^ Additionally, SBD can have a negative impact on quality of life (e.g., stigma and discomfort with ostomy) as well as lead to an increased risk of local infection and skin irritation at the ostomy site. Given these challenges, pharmacologic interventions, like maralixibat, may be particularly valuable for patients who experience breakthrough pruritus after SBD or who wish to avoid or reverse the procedure.

Intractable pruritus is one of the most common indications for liver transplant in children with ALGS, accounting for up to 69% of transplants as described by the Global Alagille Alliance (GALA) studies.^4^, ^19^ The results here suggest that the use of maralixibat could potentially reduce the need for liver transplantation in this patient population, although additional, appropriately powered, studies would be needed to confirm these findings. In fact, transplant‐free survival was significantly better in patients (who had not undergone an SBD) with ALGS treated with maralixibat compared with those in the GALA natural history cohort.^15^ Breakthrough or recurrent pruritus after SBD or its reversal may result from a variety of different factors including: recirculation of pruritogens after reversal (which may trigger pruritic symptoms), incomplete or lost diversion function (e.g., stoma closure, blockage of the diversion pathway, adaptation of the gut), or persistent hepatic production of itch mediators (which can continue especially if the liver disease progresses).^20^

Our results demonstrate that management of pruritus may be improved in patients who have already undergone SBD and experience breakthrough pruritus. Additionally, initiation of maralixibat after SBD may allow patients to undergo reversal of the procedure and ensure that their pruritus is still managed effectively. According to the US maralixibat prescribing information, the recommended dose for the treatment of cholestatic pruritus in ALGS is 380 µg/kg taken once daily.^12^ It is recommended that patients initially start at 190 µg/kg once daily and then after 1 week, increase to 380 µg/kg once daily, as tolerated.^12^ Importantly, the use of twice daily dosing in both cases was a unique situation and is not a treatment recommendation. Appropriate dosing aligned with the prescribing information and physician's recommended management algorithm should be utilized.

The approval of IBAT inhibitors, like maralixibat, has ushered in a new era in the care of patients with ALGS who have cholestatic pruritus. As IBAT inhibitors are now considered the new standard of care,^7^ newly diagnosed patients will be treated with these agents early in their clinical course. However, the path forward is less clear for patients who have previously undergone SBD. Inadequate relief of pruritus, technical complications, or cosmetic considerations may lead some patients with SBD to consider whether IBAT inhibitors would be an effective supplement to manage their uncontrolled cholestatic pruritus. As demonstrated in case 1, combination of IBAT inhibitors with biliary surgical diversion may be effective in severe cases.

## 5 CONCLUSIONS

In conclusion, treatment with maralixibat can improve outcomes in patients with ALGS who have SBD refractory pruritus, and in patients who have had a reversal of their SBD and experience refractory pruritus.

## CONFLICT OF INTEREST STATEMENT

Ryan Himes has received payments from Mirum and Ipsen related to consulting and speaking. Tamir Diamond has provided consultation for Mirum Pharmaceuticals in 2024. The remaining authors declare no conflicts of interest.

## Data Availability

All data supporting the findings of each case were included in this article. Further inquiries regarding these cases can be directed to the corresponding author.
